# Left ventricular hypertrophy in aortic stenosis: is 2-D echo reliable?

**DOI:** 10.1186/1532-429X-16-S1-P271

**Published:** 2014-01-16

**Authors:** Nicholas C Boniface, Julianne Matthews, Brandon M Mikolich, John Lisko, J Ronald Mikolich

**Affiliations:** 1 Northeast Ohio Medical University, Rootstown, Ohio, USA; 2 Sharon Regional Health System, Sharon, Pennsylvania, USA

## Background

Progressive pressure overload to the left ventricle, which occurs with aortic stenosis(AS), is a known stimulus to left ventricular hypertrophy (LVH). However, assessment of LV wall thickness with 2-D echo has failed to show a useful correlation with severity of AS. The absence of a relationship may be due to inherent limitations in 2-D echo assessment of LV wall thickness and aortic valve area. This study was designed to determine if cardiac magnetic resonance imaging (CMR) may be a more useful diagnostic tool.

## Methods

An institutional cardiac imaging database was queried to identify patients with isolated aortic stenosis. Patients with AS having both 2-D Echo and CMR studies with 6 months of each other constituted the study population. The mean LV anteroseptal and inferoposterior wall thickness measurements were computed for patients with LVH on 2-D echo, using both 2-D echo and CMR report data. 2-D echo and CMR LV wall thickness were statistically compared using a paired-sample t-test. LV wall thickness dimensions for both 2-D echo and CMR were analyzed for their statistical relationship to aortic valve area (AVA) using linear regression analysis. AVA by 2-D echo was calculated with Doppler velocity measurements using the continuity equation. AVA by CMR was measured by planimetry and confirmed with phase velocity mapping flow data.

## Results

**Figure 1:**
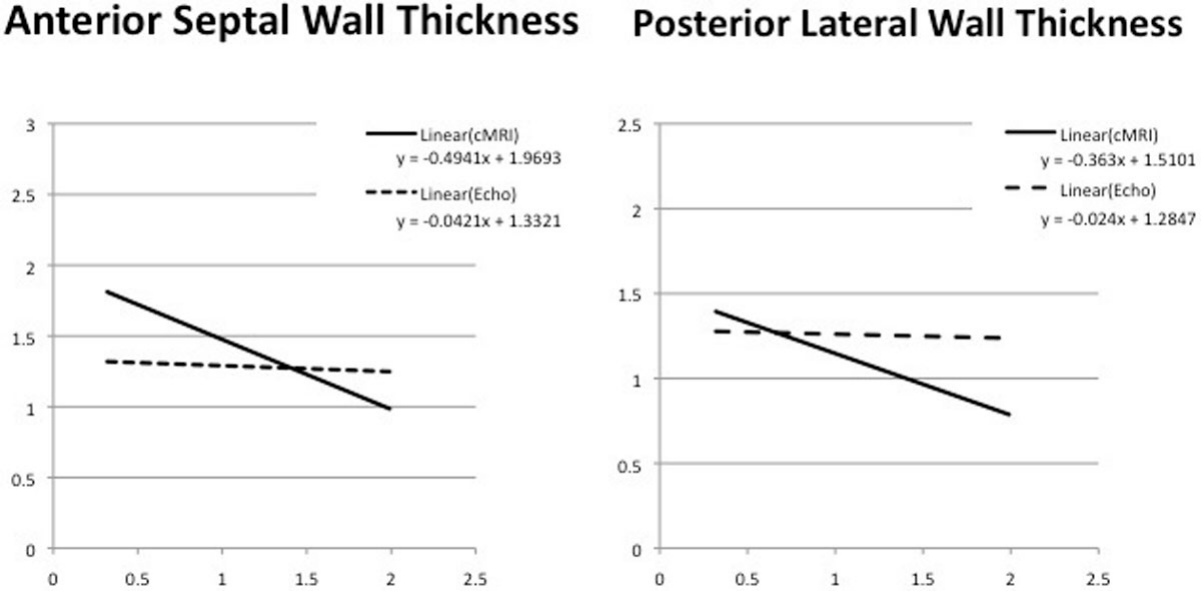
**Correlation of LV Wall Thickness and Aortic Valve Area By 2-D Echo and CMR Methods**.

111 patients with a diagnosis of AS who had both 2-D and CMR were identified. Of the 111 patients with AS, 102 had a diagnosis of LVH on 2-D echo. For AS patients with LVH on 2-D echo (n = 102) the mean anteroseptal wall thickness (ASWT) was 1.26 ± 0.20 on 2-D and 1.32 ± 0.36 on CMR, while the inferoposterior wall thickness(IPWT) was 1.24 ± 0.19 on 2-D and 1.08 ± 0.31 on CMR. Paired sample t-test revealed statistically significant difference between echo and CMR measurements for ASWT (p = 0.0498) and IPWT (p = 0.0001). The relationship of ASWT and IPWT dimensions to AVA are shown for 2-D echo and CMR in the Figure 1. Linear regression plotting shows CMR to be superior to 2-D echo for both ASWT and IPWT correlation of wall thickness and aortic valve area

## Conclusions

For patients with AS and LVH on 2-D, the 2-D echo technique appears to underestimate ASWT and overestimae IPWT relative to CMR. LV wall thickness by 2-D echo appears to have no relationship to AVA, while CMR assessments of both ASWT and IPWT appear to have a clear, but not robust, inverse relationship to AVA (see Figure 1). These data suggest LV wall thickness measurements by CMR are more useful in estimating severity of AS, than similar measurement performed with 2-D echo. These findings may be due to more accurate assessment of LV wall thickness by CMR due to better spatial resolution and/or more accurate determination of AVA by planimetry and phase velocity mapping techniques.

## Funding

None.

